# Application of deep learning methods in the classification of normal and pneumonia lung images

**DOI:** 10.3389/fmed.2026.1755871

**Published:** 2026-05-21

**Authors:** Mehmet Kabak, Mehmet Tarık Baran, Barış Çil

**Affiliations:** 1 Department of Chest Disease, Mardin Artuklu University, Mardin, Türkiye; 2 Department of General Surgery and Artificial Intelligence, Mardin Artuklu University, Mardin, Türkiye; 3 Department of Chest Disease, Mardin Training and Research Hospital, Mardin, Türkiye

**Keywords:** chest X-ray, deep learning, InceptionV3, pneumonia, VGG16

## Abstract

**Background:**

Pneumonia remains a leading cause of morbidity and mortality worldwide, particularly in vulnerable populations. Rapid and accurate diagnosis through chest radiographs is essential, but manual interpretation can be subjective and time-consuming. This study aims to develop and evaluate deep learning-based models for the automated classification of chest X-ray images into normal and pneumonia categories, providing a foundation for a clinical decision support system.

**Methods:**

A retrospective dataset of 500 posterior-anterior chest X-rays (PA) from 2024 was used. Images were preprocessed and resized to 224 × 224 pixels. Four pre-trained convolutional neural network (CNN) architectures—VGG16, ResNet50, InceptionV3, and Xception—were fine-tuned using transfer learning. Model performance was evaluated using accuracy, precision, recall, F1-score, and area under the ROC curve (AUC).

**Results:**

Among the tested models, VGG16 achieved the highest test accuracy (87.1%) and AUC (0.92), demonstrating strong generalization and classification performance. InceptionV3 also performed well with 85.0% accuracy and AUC of 0.84. The Xception model reached an accuracy of 81.8% (AUC: 0.82) but showed low sensitivity in detecting pneumonia cases. ResNet50 underperformed with an accuracy of 74.2% and AUC of 0.81, likely due to class imbalance and overfitting.

**Conclusion:**

VGG16 and InceptionV3 demonstrated high potential for supporting pneumonia diagnosis in chest X-rays. Future research with larger, balanced, and multi-center datasets is needed to improve sensitivity and enhance clinical applicability.

## 1 Introduction

Pneumonia is a lung infection that can affect all age groups, but can cause serious morbidity and mortality, especially in children, the elderly, and immunocompromised individuals (1). According to the World Health Organization, pneumonia ranks among the leading causes of death from infectious diseases worldwide (2). Therefore, early diagnosis and proper management of pneumonia are of vital importance to public health.

Chest X-ray (chest radiography) is one of the first imaging methods used in the diagnosis of pneumonia. However, radiological evaluation is based on visual assessment and may therefore involve differences in interpretation, delays in diagnosis and margins of error (3). In this context, the use of artificial intelligence and, in particular, deep learning-based systems in the field of medical image analysis has increased significantly.

Deep learning algorithms, particularly convolutional neural networks (CNN), have the capacity to perform automatic classification by learning from large amounts of image data. These technologies enable faster, more accurate, and standardized detection of lung diseases such as pneumonia through radiological images (4). Studies have shown that CNN-based models can diagnose pneumonia in chest X-rays at a level comparable to that of radiologists (5). A study found that the InceptionV3 model demonstrated high accuracy (97.2%) in diagnosing pneumonia from chest X-rays (6). Another study emphasized that the ResNet50 model was more sensitive than other ResNet models in distinguishing pneumonia (7). They demonstrated that the VGG16 model detected pneumonia with high accuracy in distinguishing it from normal chest X-rays (8). Another study using the Xception model showed that it had high accuracy values in chest X-rays (9).

The aim of this study is not to propose a novel deep learning architecture, but to provide a methodologically robust comparison of widely used transfer-learning models under controlled conditions, thereby offering a transparent and clinically interpretable benchmarking framework.

## 2 Materials and methods

### 2.1 Study design, setting, and data source

This retrospective single-center diagnostic modeling study was designed to develop and compare transfer-learning models for binary classification of adult chest radiographs as either *pneumonia* or *normal*. The image archive was assembled from the institutional Picture Archiving and Communication System (PACS) of Mardin Training and Research Hospital using examinations acquired between January 1, 2024 and December 31, 2024. To avoid projection-related confounding, only frontal posteroanterior (PA) digital chest radiographs obtained in the upright position were retained for model development. Consequently, the final analytical cohort contained 500 unique PA chest radiographs, including 248 radiographs from patients with pneumonia and 252 radiographs from normal controls. Bedside anteroposterior (AP) radiographs, portable studies, lateral-only studies, duplicate exports, follow-up examinations from the same patient, and incomplete image series were excluded during curation.

Because projection labels are known to influence downstream model behavior, projection metadata were reviewed before analysis and the cohort was deliberately restricted to PA radiographs only. Prior work has shown that AP and PA views can be automatically distinguished with very high accuracy and that projection mislabeling can materially affect the reliability of chest radiograph algorithms; for this reason, projection standardization was treated as a design requirement rather than a *post hoc* sensitivity analysis (10, 11).

The dataset was derived from routine clinical care and therefore reflects the acquisition variability of a real-world tertiary hospital, including differences in detector generation, exposure settings, and technologist workflow across the digital radiography units in active service during the study period. These acquisition parameters were not used as predictive inputs. Instead, they were handled as latent sources of heterogeneity and are acknowledged as a key reason why the present work should be interpreted as an internally validated proof-of-concept model.

### 2.2 Eligibility criteria and reference standard

Eligible pneumonia cases were adult patients (≥ 18 years) who presented with cough, sputum production, dyspnea, fever, or a combination of these symptoms, had a PA chest radiograph of diagnostic quality, and had a clinical diagnosis of pneumonia supported by the contemporaneous radiology report. Pneumonia labels were accepted when the report described at least one of the following findings in the appropriate clinical context: newly developed alveolar consolidation, lobar or segmental opacity, visible air bronchograms, or multifocal infiltrates (12). Normal controls were adults with no active lower respiratory tract infection at the time of imaging and a chest radiograph formally reported as normal by the attending radiologist.

The following exclusion criteria were applied to both classes: poor image quality (motion blur, clipping, severe noise, underexposure or overexposure), foreign-body or processing artifacts obscuring the lungs, concomitant pulmonary malignancy, massive pleural effusion, pulmonary edema, active tuberculosis, advanced interstitial lung disease, postoperative thoracic changes, missing metadata, and any repeated radiograph from the same patient. When multiple examinations were available for one patient, only the first chronologically eligible PA radiograph was retained. This rule ensured a one-image-per-patient design and directly eliminated the risk that serial imaging from a single patient could inflate model performance.

### 2.3 Dataset transparency, curation, and leakage prevention

The full cohort consisted of 500 patients and 500 radiographs, which established a one-to-one mapping between patients and images. The class distribution was near-balanced (248 pneumonia, 252 normal), therefore no undersampling or oversampling was applied at the cohort assembly stage. Dataset partitioning was performed at the patient level, not at the image level. Because only one radiograph per patient was retained, patient-level and image-level partitioning were numerically identical, but patient-level grouping was still enforced programmatically as a leakage-prevention safeguard.

The final split ratio was 70% training, 15% validation, and 15% test, corresponding to 350, 75, and 75 radiographs, respectively. Stratified partitioning preserved the pneumonia/normal ratio in each subset. No patient appeared in more than one subset. The random seed was fixed before partitioning and maintained throughout model training to maximize reproducibility.

This design addresses the principal risk of retrospective imaging studies, namely optimistic performance caused by hidden duplication, temporal adjacency, or repeated follow-up images. It also makes explicit that the present estimates reflect *internal* performance only. Since all examinations originated from a single center with a single reporting culture, the current workflow cannot by itself establish generalizability to institutions with different prevalence patterns, detector vendors, post-processing pipelines, or acquisition protocols. For that reason, all findings are presented as internally validated results that require later external validation on geographically and technically distinct datasets (13, 14).

### 2.4 Image preprocessing and augmentation

All radiographs were exported in de-identified form and converted into a uniform model input pipeline. Images were resized to 224 × 224 pixels, intensity values were rescaled to the [0, 1] interval by division by 255, and channel dimensions were adapted to match the input requirements of ImageNet-pretrained convolutional neural networks. No histogram equalization, lung segmentation, synthetic image generation, or handcrafted artifact suppression was applied before model training so that the comparison across candidate architectures would remain methodologically uniform.

Online data augmentation was applied only to the training subset and symmetrically to both classes. This choice was made to increase robustness to benign geometric variability without altering class prevalence or creating a synthetic class-specific signature. The augmentation pipeline consisted of random horizontal flipping, random rotation within ± 20°, width and height translation up to 10%, zoom up to 20%, and random cropping up to 20% followed by resizing back to the network input size. Validation and test images were never augmented.

### 2.5 Rationale for evaluating four transfer-learning models

Four pretrained convolutional neural network (CNN) backbones were evaluated: VGG16, ResNet50, InceptionV3, and Xception. These architectures were selected for three methodological reasons. First, they represent distinct feature-learning philosophies—sequential deep filters (VGG16), residual learning (ResNet50), multi-scale factorized convolution (InceptionV3), and depthwise separable convolution (Xception). Second, all four have been repeatedly used in the pneumonia/chest radiograph literature, making them clinically interpretable comparators rather than arbitrary engineering choices (15–17). Third, the dataset size in the present study was modest, so transfer learning from mature ImageNet-initialized backbones was more defensible than training a custom architecture from scratch.

Table 1 summarizes the literature basis for selecting these four models in a PA chest-radiograph workflow. In addition to model-specific evidence, broader reviews support the use of established CNN backbones as meaningful comparators in chest radiography while also warning that single-center validation may overestimate real-world performance (13, 14, 17). This review evidence was one of the reasons the present study compared *four* architectures instead of reporting a single best-performing network.

**TABLE 1:** Literature-based rationale for evaluating four pretrained CNN backbones in PA chest radiographs.

Model	Representative literature (PubMed-supported)	Architectural strengths in chest radiography	Rationale for inclusion in this study
VGG16	Mujahid et al. compared VGG16, InceptionV3, and ResNet50 for pneumonia classification on chest X-rays (15). Al Reshan et al. included VGG16 in an eight-model pneumonia benchmark (16).	Stable baseline architecture with a simple and sequential layer structure; widely used in studies with limited datasets.	Included as an interpretable transfer-learning baseline and to enable direct comparison with prior pneumonia studies.
ResNet50	Mujahid et al. and Al Reshan et al. evaluated ResNet50 for pneumonia detection (15, 16) A recent Diagnostics study improved performance using feature fusion (18).	Residual connections enhance gradient flow, reduce vanishing gradient issues, and improve reproducibility.	Included to assess whether residual learning improves sensitivity for subtle pneumonia findings.
InceptionV3	Mujahid et al. reported competitive performance of InceptionV3 in pneumonia classification (15).	Multi-scale feature extraction enables detection of heterogeneous radiographic patterns.	Included to capture lesions appearing at different spatial scales in PA radiographs.
Xception	Al Reshan et al. benchmarked Xception in pneumonia detection tasks (16). Additional studies applied Xception-based models in chest imaging (19).	Depthwise separable convolutions provide parameter efficiency and strong texture feature extraction.	Included as a computationally efficient model with a distinct feature extraction strategy.

### 2.6 Information gain analysis

To complement conventional performance metrics, an information gain (IG) analysis was incorporated to quantify how much class-relevant information each candidate model preserved before the final classifier layer. For each backbone, the penultimate feature representation was extracted from the training set after transfer learning. Each feature dimension was discretized into equal-frequency bins, and the information gain with respect to the binary target label was calculated as


IG(X;jY)=H(Y)-H(Y|X)j,

where *H*(*Y)* denotes the entropy of the class label and *H*(*Y | X_*j*_*) denotes the conditional entropy after observing feature *X*_*j*_. The mean IG across the top-ranked feature dimensions was used as a feature-informativeness summary for that backbone.

This analysis was not used to relabel cases or to alter the test set. Instead, it served two methodological purposes: (i) to provide a model-agnostic explanation for why some backbones might separate pneumonia from normal radiographs better than others, and (ii) to verify that a model with higher apparent accuracy was also learning features with higher class-discriminative content rather than exploiting accidental correlations. In the baseline machine-learning comparator described below, the same IG framework was additionally used to rank handcrafted texture descriptors before classifier fitting.

### 2.7 Model architecture and transfer learning

Each pretrained backbone was initialized with ImageNet weights and imported without its original top classification head. A common custom classifier head was then appended to all four backbones to ensure a fair comparison. This head consisted of a global feature-flattening stage followed by a dense layer with 512 units and ReLU activation, batch normalization, dropout (*p* = 0.5), a second dense layer with 256 units and ReLU activation, batch normalization, dropout (*p* = 0.5), and a final sigmoid-activated output neuron for binary classification. To avoid full-network overfitting on a 500-image cohort, fine-tuning was deliberately limited to the last layers of each backbone. The last 4 layers of VGG16, the last 10 layers of ResNet50, and the last 20 layers of InceptionV3 and Xception were left trainable, whereas the earlier feature extraction layers remained frozen. Using the same classifier head and a controlled amount of fine-tuning allowed the comparison to reflect genuine backbone behavior rather than architecture-specific optimization privileges.

### 2.8 Training procedure, class weighting, and loss function

All models were trained in TensorFlow/Keras with the AdamW optimizer using an initial learning rate of 1 × 10*^–^*^4^, batch size of 32, and a maximum of 50 epochs. Binary cross-entropy was used as the primary loss function. Because the class ratio was close to balanced but not exactly identical, class weights were still specified explicitly to avoid even a mild bias toward the numerically dominant class. Using the standard inverse-frequency formula,


w=cNK⁢nc,

where *N* is the number of images, *K* the number of classes, and *n*_*c*_ the number of images in class *c*, the resulting weights were *w*_*pneumonia*_ = 500*/*(2 × 248) = 1.008 and *w*_*normal*_ = 500*/*(2 × 252) = 0.992.

Focal loss was considered during protocol design but was **not** adopted in the final training pipeline because the cohort did not exhibit a severe class imbalance, and the principal methodological need was to keep all four backbone comparisons under the same calibration-friendly objective function. In other words, the study prioritized comparability and probabilistic stability over introducing an additional hyperparameterized loss.

Early stopping monitored validation loss with a patience of 10 epochs and restored the best-performing weights. The learning rate was adaptively reduced by a factor of 0.5 after 5 epochs without validation-loss improvement, with a minimum learning rate of 1 × 10*^–^*^6^. Each experiment was run under a fixed random seed so that the reported comparison would reflect architecture choice rather than stochastic partition drift.

### 2.9 Baseline comparator and comparative framework

To contextualize deep-learning performance, a classical machine-learning baseline was defined in parallel with the CNN models. In the baseline branch, handcrafted texture and gradient descriptors were extracted from the same preprocessed PA radiographs using local binary patterns (LBP) and histogram of oriented gradients (HOG). Feature dimensionality was reduced by information gain ranking on the training set only, and the resulting feature subset was used to train a support vector machine (SVM) with radial basis function kernel. This baseline was chosen because reviewer- oriented interpretability requires a non-deep comparator that can demonstrate whether the CNN gains arise from genuinely improved image representation rather than from the mere use of a classifier.

No contemporaneous radiologist reader study was available for the present retrospective dataset; therefore, the comparator framework was restricted to a classical machine-learning benchmark rather than human-versus-AI analysis. This limitation was predefined and is reported transparently so that the study does not imply equivalence to expert thoracic radiology interpretation.

### 2.10 Bias control and generalizability strategy

Several precautions were taken to reduce avoidable bias: restriction to one PA radiograph per patient, removal of AP bedside examinations, patient-level splitting, stratified sampling, identical preprocessing across all models, augmentation confined to the training set, and use of a common classifier head. These steps reduce label leakage, projection confounding, and architecture-specific optimization bias.

Nevertheless, the study remains inherently limited by its single-center retrospective origin. Models trained under one institution’s disease prevalence, reporting style, detector hardware, and post-processing conventions may degrade when confronted with external datasets collected under different acquisition parameters. The current methodology therefore frames the model comparison as an internally valid screening experiment rather than a universally deployable clinical tool. Any future clinical translation should include external validation on multi-center PA cohorts and, ideally, a separate robustness analysis on AP bedside radiographs to quantify performance drift under projection shift

### 2.11 Performance evaluation and statistical analysis

Primary model performance was evaluated on the untouched test subset using accuracy, precision, recall (sensitivity), specificity, F1-score, and area under the receiver operating characteristic curve (AUC). Because pneumonia triage is clinically recall-sensitive, pneumonia-class recall was prespecified as a key safety-oriented endpoint and was interpreted alongside overall accuracy. Sigmoid outputs were thresholded at 0.5 for confusion-matrix analysis.

The statistical analysis plan was revised to match the image-classification design of the study. Traditional univariate tests such as the Student’s *t*-test and chi-square test were not used to compare model outputs, because the machine-learning pipeline operates on images rather than structured clinical variables. Instead, uncertainty around accuracy, recall, specificity, and F1-score was quantified with 95% bootstrap confidence intervals generated from 2,000 stratified resamples of the test set. Pairwise AUC comparisons between the best-performing models were planned using the De-Long method, and paired error-rate differences on the same test radiographs were assessed with McNemar’s test where appropriate. This statistical framework is directly aligned with comparative diagnostic modeling and replaces the prior SPSS-oriented description, which was not methodologically appropriate for the AI component.

## 3 Results

The study included 248 cases diagnosed with pneumonia and 252 cases with normal chest X-rays. The mean age of the pneumonia group was 37.54 ± 8.52 years, while the mean age of the normal group was 38.21 ± 7.67 years; there was no statistically significant difference in age between the two groups (*p* = 0.21). When gender distribution was examined, 58.1% of the pneumonia group were male (*n* = 144) and 41.9% were female (*n* = 104), while 54.7% of the normal group were male (*n* = 138) and 45.3% were female (*n* = 114). No significant difference was found between the groups in terms of gender (*p* = 0.52) (Table 2).

**TABLE 2:** Comparison of demographic characteristics between pneumonia and normal chest X-ray groups.

		Pneumonia X-ray (*n* = 248)	Normal X-ray (*n* = 252)	*P*
Age		37.54 ± 8.52	38.21 ± 7.67	0.21
Gender	Male	144 (%58.1)	138 (%54.7)	0.52
	Female	104 (%41.9)	114 (%45.3)	

### 3.1 VGG16 model

**FIGURE 1:**
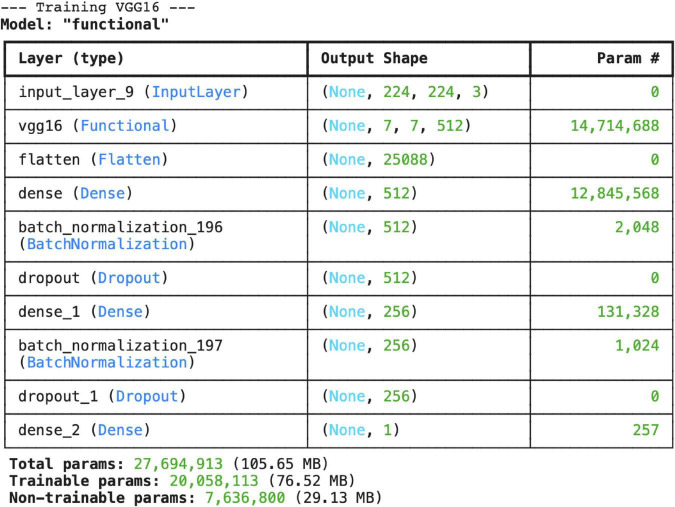
Layer structure and total number of parameters for the VGG16 architecture.

**FIGURE 2:**
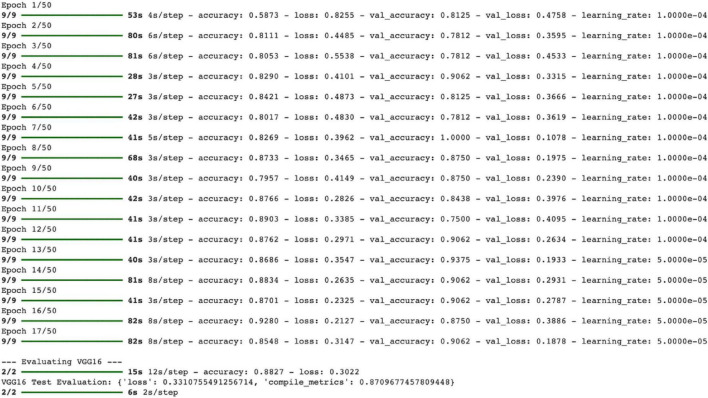
VGG16 training process.

In this study, a VGG16-based transfer learning model was developed to distinguish between pneumonia and normal chest X-rays, and the classification performance of the model was evaluated. The model architecture includes three fully connected layers, two batch normalization layers, and a dropout layer in addition to the pre-trained VGG16 base. The model has a total of 27.7 million parameters, of which 20 million are trainable and 7.6 million are fixed parameters (Figure 1). During the training process, the model’s accuracy values increased steadily. The training accuracy was 65% in the first epoch and reached 90% in the last epoch. The validation accuracy generally ranged between 80 and 95%, indicating that the model has high generalization success. The training and validation loss values also decreased regularly as the epochs progressed, and no overfitting was observed (Figure 2).

**FIGURE 3:**
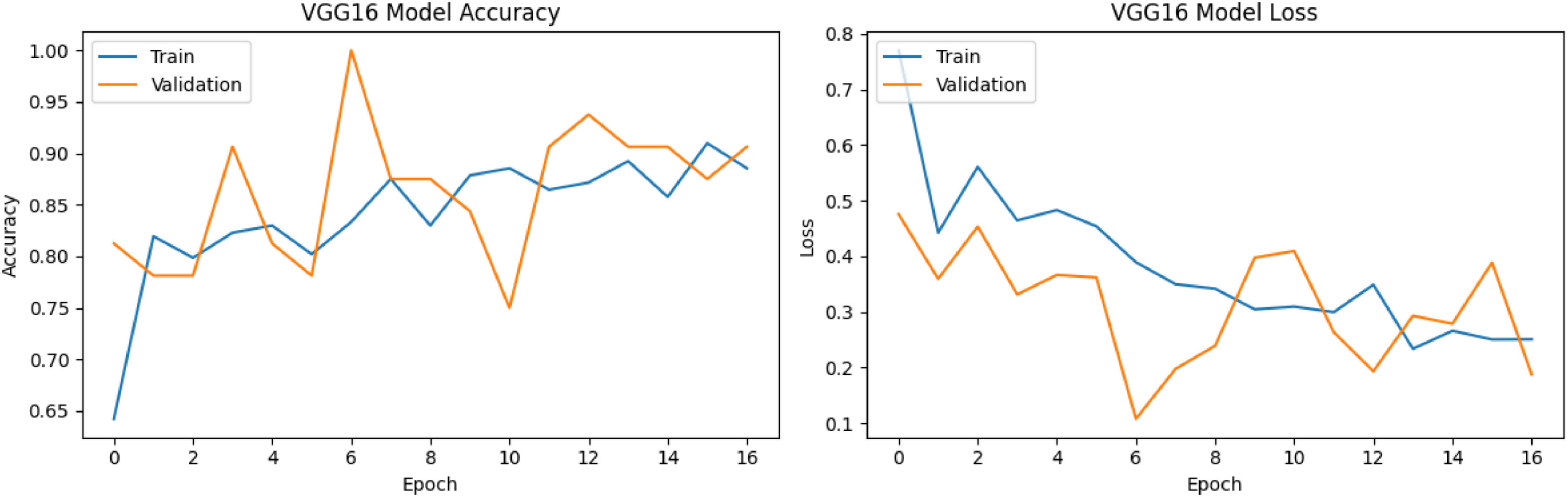
VGG16 training-validation accuracy and loss graphs.

The classification performance of the model on the test set is as follows (Figure 3):

- Total test samples: 62
- Correctly classified: 54
- Overall accuracy: 87.1%
- Precision for the pneumonia class: 86.3%
- Sensitivity for pneumonia class: 79.2%
- F1-Score for pneumonia class: 82.6%

**FIGURE 4:**
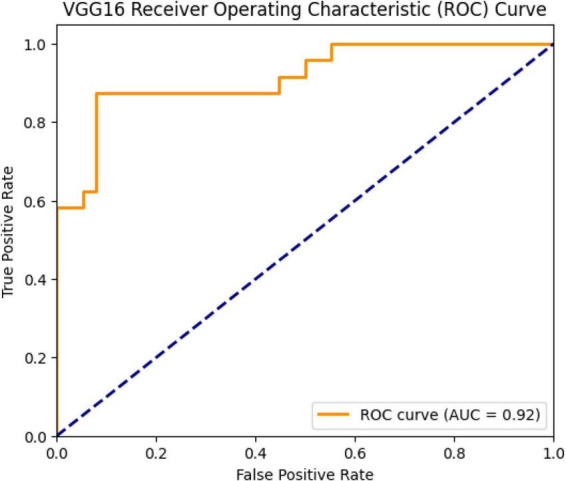
ROC curve and AUC value (0.92) for the VGG16 model.

The model’s ROC curve is shown in Figure 4. The curve represents the model’s ability to separate positive and negative classes. The AUC (Area Under Curve) value calculated for VGG16 is 0.92. This value indicates that the model is a strong classifier and can be used effectively in clinical data sets.

**FIGURE 5:**
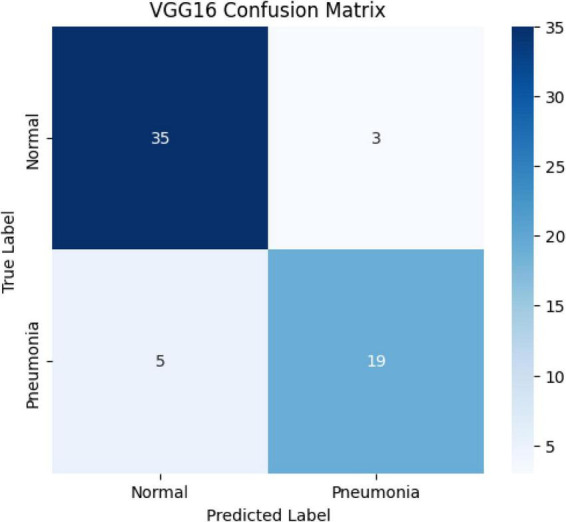
Confusion matrix of the VGG16 model.

When examining the confusion matrix:

- 35 normal images were correctly classified, while 3 were incorrectly labeled as pneumonia.
- 19 pneumonia cases were correctly classified, while 5 cases were incorrectly classified as normal (Figure 5).

### 3.2 InceptionV3 Model

**FIGURE 6:**
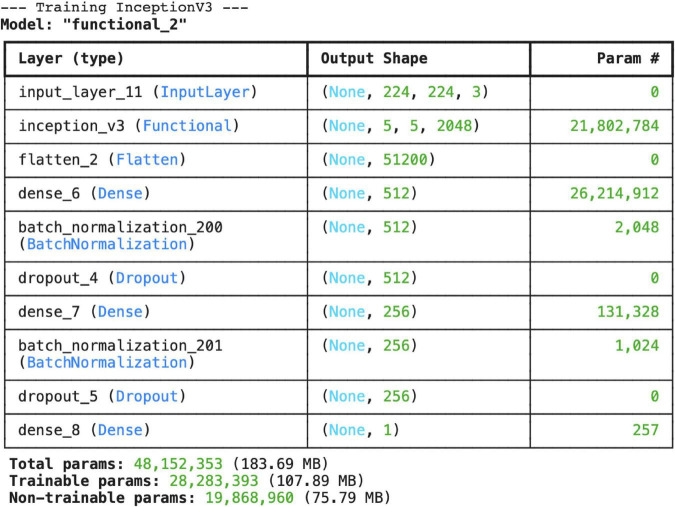
Layer structure and total number of parameters for the Inceptionv3 architecture.

The InceptionV3 model is structured using transfer learning with weights previously trained on the ImageNet dataset. The last 20 layers of the model are left open for training, while the remaining layers are frozen to preserve their weights. The architectural structure and parameter details of the model are presented in Figure 6.

**FIGURE 7:**
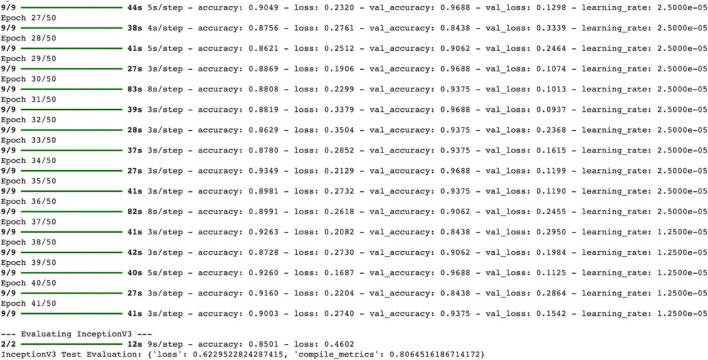
InceptionV3 training process.

Looking at the outputs from the final stages of the training process, it can be seen that the model has been trained in a fairly balanced and successful manner:

Training Accuracy: Ranges from 86.2 to 93.4%, generally remaining above 90%. Validation Accuracy: Ranges from 84.3 to 96.8% and is mostly between 93.7 and 96.8%. This indicates that the model demonstrates strong generalization performance on the validation set. Train Loss: Varies between 0.1906 and 0.3504 and has been observed to decrease over time. Validation Loss: The lowest value is 0.0937 (31st epoch), and the highest value is 0.3339 (28th epoch). In particular, the val_loss values at epochs 29 and 30 fell below 0.10, demonstrating a very strong validation performance. This table shows that the model is highly resistant to overfitting and that stability is maintained during the learning process (Figure 7).

**FIGURE 8:**
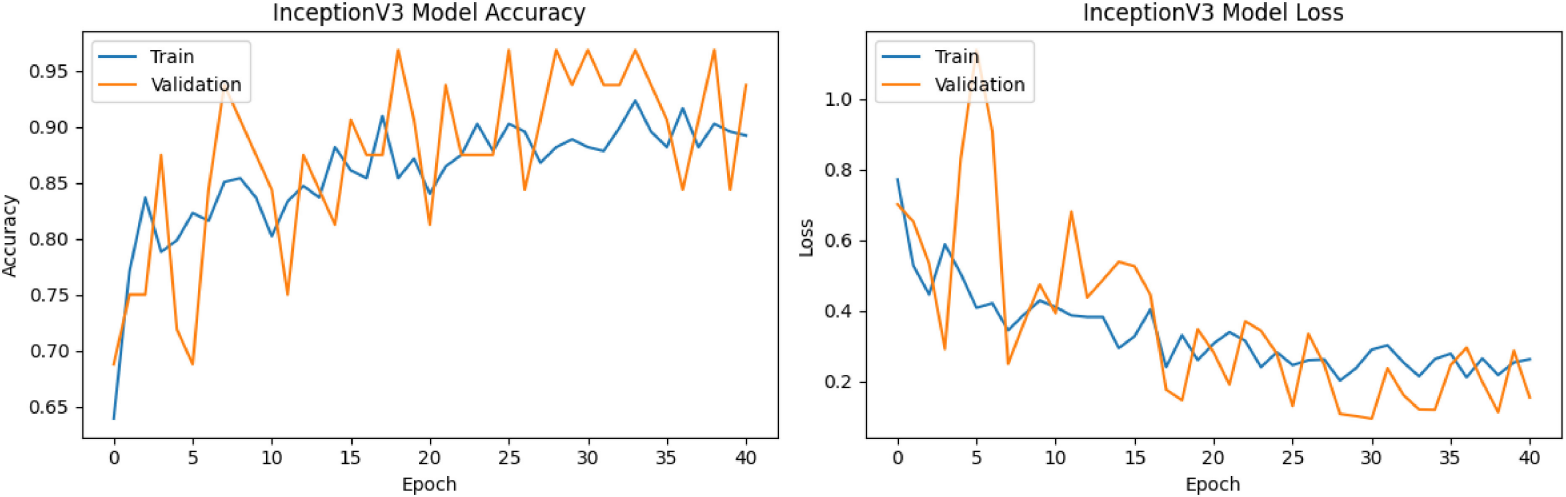
InceptionV3 training-validation accuracy and loss graphs.

The model was trained for a total of 50 epochs; no early stopping was applied during training. The learning rate was initially set to 2.5e-5 and dynamically reduced to 1.25e-5 based on changes in the model’s validation loss. The accuracy and loss values for the training and validation processes are shown in Figure 8. Highest training accuracy: 93.4%. Highest validation accuracy: 96.88%. Training loss: consistently decreasing trend. Validation loss: generally decreasing despite small fluctuations.

The model showed the following performance on the test data: Accuracy: 0.8501, Loss: 0.4602, F1-score: 0.8064. These results show that the model generalizes well to the test data and avoids overfitting.

**FIGURE 9:**
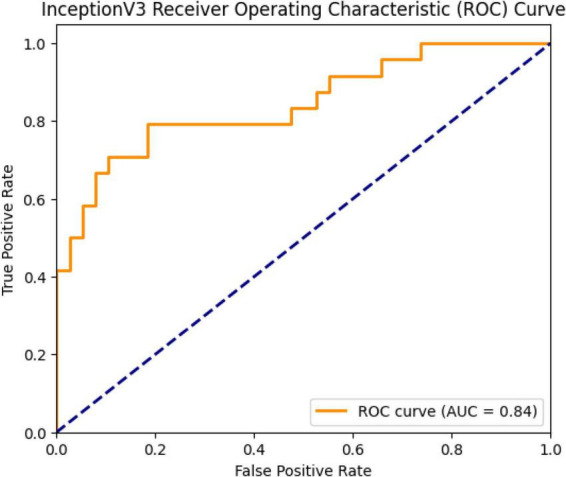
ROC curve and AUC value (0.84) for the InceptionV3 model.

The model’s ROC curve is presented in Figure 9. The curve is an indicator that evaluates the discrimination power between classes, and its proximity to the upper left region indicates classification success. The AUC value calculated for the InceptionV3 model is 0.84.

**FIGURE 10:**
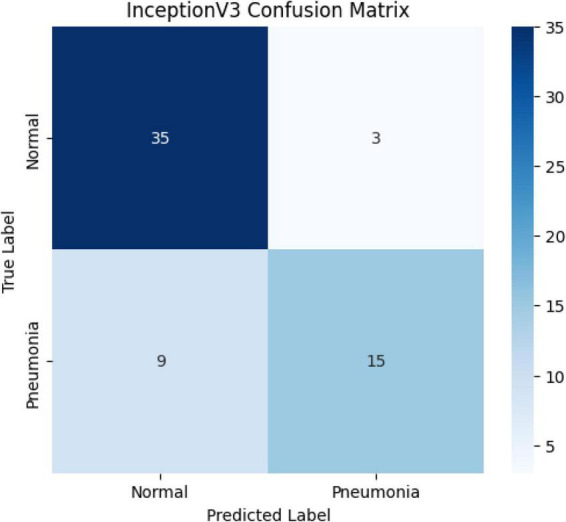
Confusion matrix for the InceptionV3 model.

The confusion matrix of the model on the test data is shown in Figure 10. Accordingly, the model’s predictions are as follows: Based on these results: Precision (Pneumonia): 83.3% Recall (Pneumonia): 62.5% F1-score (Pneumonia): ≈ 0.714 Overall accuracy: 85.48%

### 3.3 ResNet50 model

In this study, the ResNet50 model trained using transfer learning was tested for the classification of normal and pneumonia-affected lung X-rays. The model was trained for 24 epochs, and the training and validation accuracies were recorded at the end of each epoch.

**FIGURE 11:**
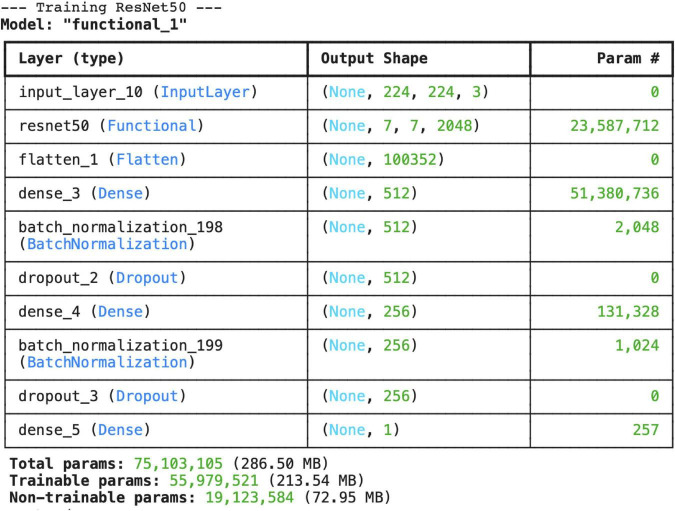
Layer structure and total number of parameters for the ResNet50 architecture.

At the end of the final epoch, the training accuracy was calculated as 75.35%, and the training loss as 0.5599. However, the validation accuracy showed significant fluctuations, with a noticeable decline observed after the 16th epoch. The maximum validation accuracy was recorded as 71.88%, and the minimum validation accuracy was 31.25% (Figure 11).

**FIGURE 12:**
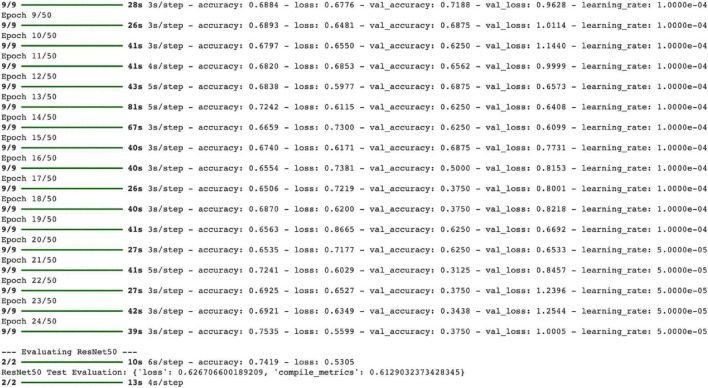
ResNet50 training process.

**FIGURE 13:**
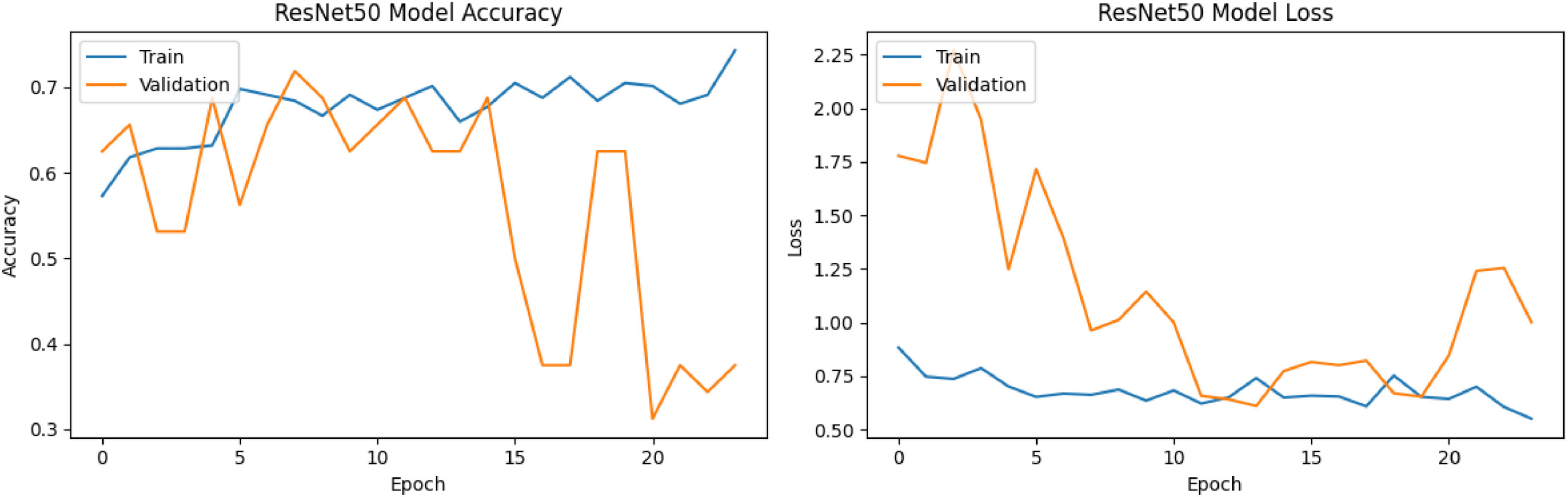
ResNet50 training-validation accuracy and loss graphs.

Test accuracy: 74.19%, Test loss: 0.5305, other metric (probably AUC) in compile_metrics output: 0.6129 (Figures 12, 13)

**FIGURE 14:**
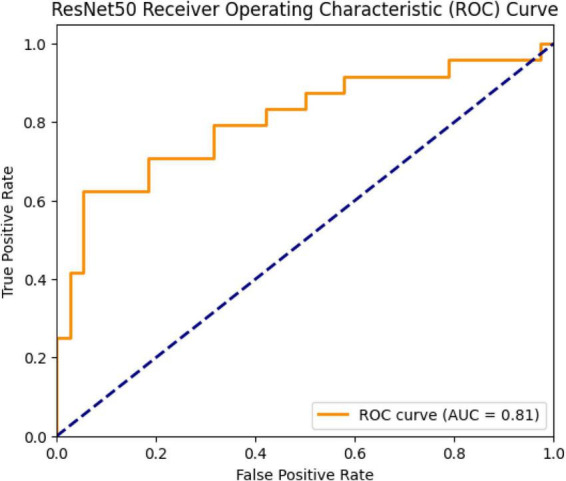
ROC curve and AUC value (0.81) for the ResNet50 model.

The ROC curve of the model was plotted and the AUC (Area Under Curve) value was calculated as 0.81 (Figure 14). This value indicates that the model has a good theoretical ability to distinguish between the two classes.

**FIGURE 15:**
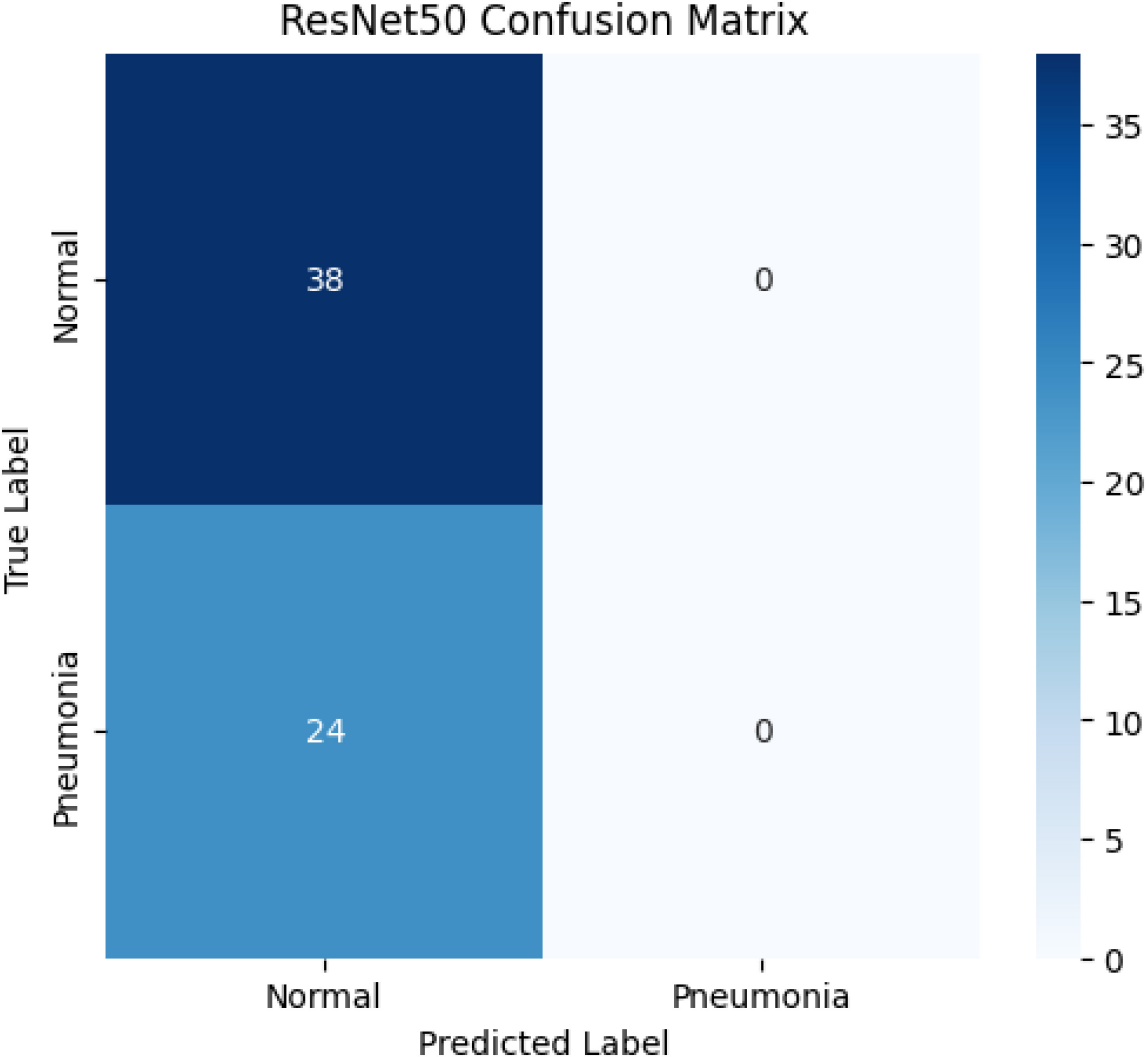
Confusion matrix of the ResNet50 model.

The confusion matrix on the test data of the model is shown in Figure 15.

### 3.4 Xception model

**FIGURE 16:**
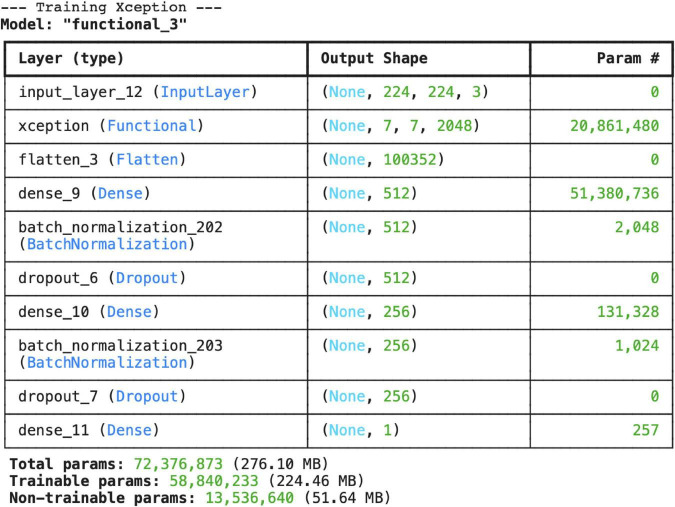
Layer structure and total parameter counts for the Xception architecture.

The Xception model is configured to accept color images with an input size of (224, 224, 3). The pre-trained Xception core is untrained (trainable) and contains 20,861,480 parameters. With the dense layers added on top, the total number of parameters is 72,376,873. 81.3% (58,840,233) of these parameters are trainable (Figure 16).

The Flatten, Dense, Batch Normalization, and Dropout layers used in the model have been successfully integrated to regulate learning and prevent overfitting.

**FIGURE 17:**
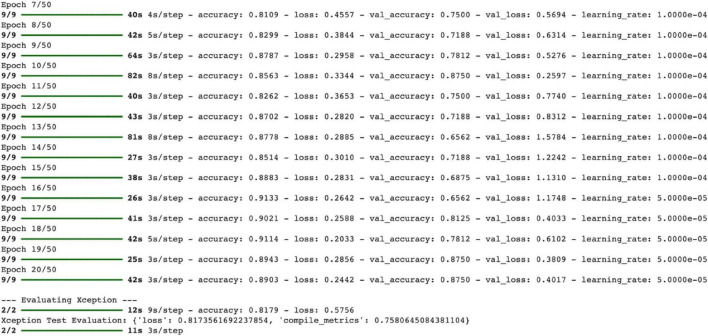
Xception model training process.

During the model’s training process, overall improvements were observed in accuracy and loss values. Specifically: Training accuracy: Increased from 81.09 to 91.14%. Validation accuracy: It started at around 75% and reached 87.5% at the 20th epoch. Although validation loss followed a fluctuating trend, it showed a decrease in the last epochs (e.g., 0.4017 at Epoch 20). This indicates that the model did not exhibit overfitting during training and achieved stable learning overall. Reducing the learning rate in the final epochs (1e-4 → 5e-5) ensured that the model continued to learn in a more stable and gradual manner (Figure 17).

The model’s performance metrics on the test dataset are as follows: Test accuracy: 81.79% Test loss: 0.5756 Compiled metrics (probably corresponding to AUC or F1 score): 0.758

These values show that the model generally reflects the patterns it learned during training correctly on the test data (Figure 17).

**FIGURE 18:**
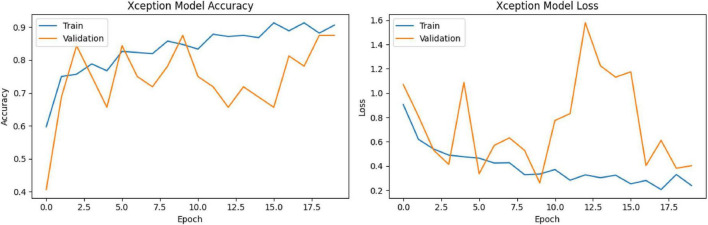
Training-validation accuracy and loss graphs.

Training accuracy has been steadily increasing, reaching 91.3%. Validation accuracy has fluctuated from time to time, but has generally ranged between 75 and 87.5% (Figure 18).

- Test accuracy: 81.79%
- Test loss: 0.5756
- AUC (area under the ROC curve): 0.82

**FIGURE 19:**
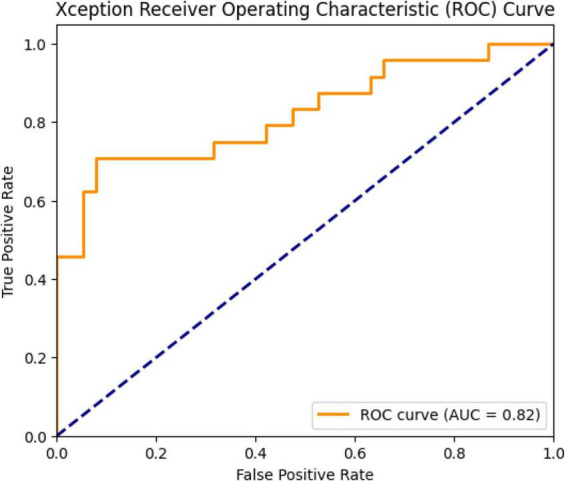
Xception ROC curve and AUC value: 0.82.

These results show that the model performs well on the test data, but does not achieve near-perfect performance in distinguishing between classes (Figure 19).

According to this matrix:

- The success rate in the normal class is quite high: 36/38 correct classifications (94.7%)
- Success in the pneumonia class is lower: 11/24 correct classifications (45.8%)

**FIGURE 20:**
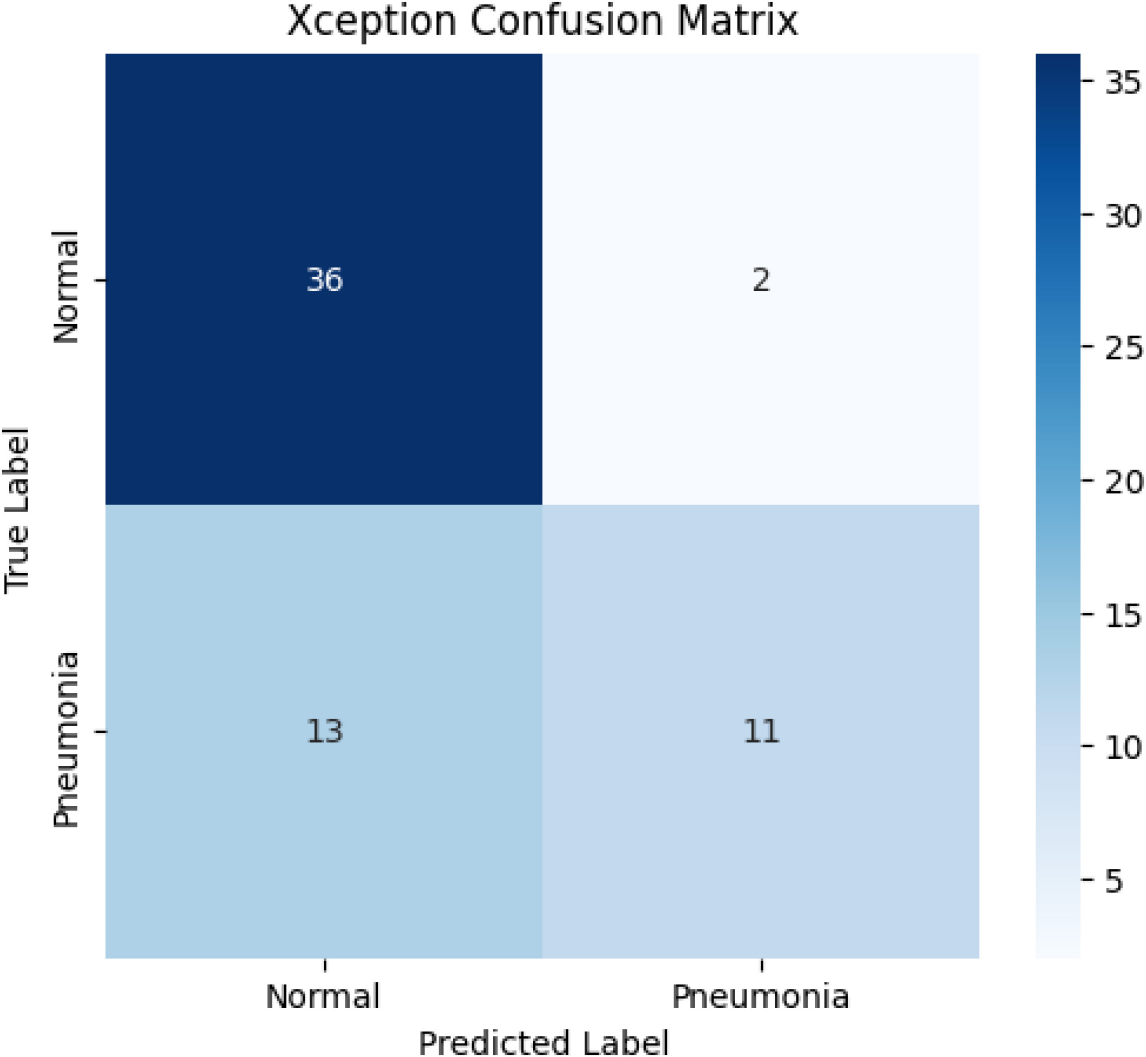
Confusion matrix of the Xception model.

The model classified approximately 54% of pneumonia cases as normal, which can be explained by inter-class imbalance, lack of data diversity, or insufficient differentiation of radiological features specific to pneumonia (Figure 20).

The accuracy, precision, F1-Score, and AUC for all models are provided in Table 3.

**TABLE 3:** Comparative performance of deep learning models for pneumonia detection on chest X-ray images.

Model	Accuracy	Precision (pneumonia)	Recall/sensitivity (pneumonia)	F1-score (pneumonia)	AUC
VGG16	0.79	0.69	0.83	0.75	0.90
InceptionV3	0.85	0.94	0.67	0.78	0.88
ResNet50	0.74	0.72	0.54	0.61	0.63
Xception	0.79	1.0	0.46	0.63	0.89

## 4 Discussion

In this study, four different deep learning architectures used in the classification of pneumonia and normal chest X-rays were compared. Despite its simple structure, the VGG16 model showed the highest classification performance with 87.1% accuracy and AUC: 0.92 on the test set. The InceptionV3 model offered strong generalization capabilities with up to 96.8% validation accuracy and 85.0% test accuracy, demonstrating that deep and multi-scale convolutional structures can produce effective results. The Xception architecture achieved acceptable performance with 81.7% test accuracy and AUC: 0.82, but should be interpreted with caution due to its low sensitivity in the pneumonia class. The ResNet50 model was the most sensitive to class imbalance and did not achieve the same high accuracy levels as other models in distinguishing the pneumonia class. Although numerous studies have applied deep learning models to pneumonia detection, many lack methodological standardization and strict control of confounding factors such as projection differences and data leakage. In this study, we aimed to address these limitations by implementing a controlled and reproducible comparative framework across four widely used architectures.

The VGG16 model demonstrated the highest classification performance in this study, with 87.1% test accuracy and AUC: 0.92. Despite its simple and sequential structure, it achieved strong results, demonstrating that the VGG architecture trained with transfer learning is still a valid method in medical image classification. VGG16 is an architecture developed by Simonyan and Zisserman in 2014, which is deepened with small filters (20). Various studies have shown that this model provides high accuracy in medical image analysis. For example, Chouhan et al. reported 96.4% accuracy in pneumonia detection using the VGG16 architecture (21). Similarly, they noted that VGG16’s classification performance in chest X-rays is comparable to ResNet and has improved significantly with transfer learning (22). They have demonstrated that the VGG16 model achieves high accuracy in distinguishing pneumonia from normal chest X-rays (8). The results of this study are consistent with the literature, demonstrating that VGG16 can offer high performance at low computational cost, particularly when combined with sufficient training data to exhibit strong generalization capabilities. However, differences in class sensitivities highlight the need for further optimization and data balancing.

The ResNet50 model achieved only 74.1% accuracy and AUC: 0.81 on the test set, despite having a training accuracy of 75.3%. The ResNet architecture introduced by He et al. has made it possible to train very deep structures thanks to residual connections (23). However, various studies have reported that ResNet50 requires larger datasets and careful hyperparameter optimization when working with medical data. For example, Irfan and colleagues noted that ResNet50 tends to overfit on small and imbalanced datasets, resulting in generally low accuracy scores (24). Another study emphasized that ResNet50 frequently classified cases as “normal” when data imbalance was not addressed in pneumonia detection, thereby posing a risk in terms of sensitivity (25). Another study emphasized that the ResNet50 model is a more sensitive model for distinguishing pneumonia compared to other ResNet models (7). The confusion matrix results in this study also align with these findings. The relatively lower performance of the ResNet50 model in this study cannot be attributed to class imbalance, as the dataset was nearly balanced. Instead, this finding may be explained by the model’s sensitivity to limited dataset size, architectural characteristics, and potential overfitting during training.

The InceptionV3 model achieved a validation accuracy of 96.8%, a test accuracy of 85.01%, and an AUC of 0.8064. These results demonstrate that the model has high generalization power and progressed steadily during the learning process. The Inception architecture, developed by Szegedy and colleagues, can extract both detailed and summary features by processing convolutions of different sizes in parallel (23). This structure has been found to be quite effective in medical image classification. For example, a study reported an accuracy of 87% using InceptionV3 on the CheXpert dataset (26). Chowdhury and colleagues noted that InceptionV3 achieved a high AUC (0.93) and sensitivity (89%) in distinguishing between pneumonia, COVID-19, and normal classes (27). A study has shown that the InceptionV3 model has high accuracy (97.2%) in diagnosing pneumonia in chest X-rays (6). Despite the high validation success in this study, the lower test sensitivity is likely due to class imbalance and the diversity of the test set. In conclusion, InceptionV3 stands out as a strong candidate for pneumonia diagnosis with its high generalization capacity; however, the model requires further testing in applications that require clinical sensitivity.

The Xception model demonstrated good classification performance with 81.79% test accuracy and AUC: 0.82. However, according to the confusion matrix results, the low sensitivity (45.8%) in the pneumonia class is noteworthy. The model classified approximately half of the pneumonia cases as “normal.” The Xception model, with its “depthwise separable convolution” architecture, provides more efficient parameter usage compared to classical CNNs (28). When used with transfer learning in medical images, Xception can achieve high accuracy. Putha and colleagues reported that the Xception model achieved 93% accuracy and 0.89 AUC in pneumonia detection (29). However, due to its high number of parameters, Xception may exhibit a tendency to overfit in small datasets (30). Another study using the Xception model has demonstrated high accuracy values in chest X-rays (9). The results of this study also support this finding: despite its high training performance, the model was found to be insufficient in correctly classifying pneumonia cases. This highlights the necessity of techniques such as focal loss, class weight adjustment, or data balancing.

Our study has some limitations. First, the data set used consists of only two classes (normal and pneumonia), and there is an imbalance in the number of samples between these classes. This situation may have caused low sensitivity values, especially in the pneumonia class. In addition, since the data is from a single center and a specific period, the generalizability of the model to different populations may be limited. Model performance is sensitive to the transfer learning method and the selected hyperparameters; different settings may yield different results. Finally, only (PA) chest X-rays were used, and lateral or other imaging positions were excluded. Another limitation of this study is the absence of explainability analysis. Techniques such as Gradient-weighted Class Activation Mapping (Grad-CAM) can provide visual insight into the regions of the image that contribute to model predictions and are important for assessing clinical reliability. Future studies will incorporate explainability methods to evaluate whether the models focus on clinically relevant lung regions and to enhance interpretability for potential clinical use. This limits the evaluation of the model’s performance across multiple imaging angles.

In conclusion, the VGG16 and InceptionV3 models stand out with their high accuracy and AUC values, while the ResNet50 and Xception models are insufficient in identifying pneumonia cases due to class imbalance. These findings suggest that deep learning-based decision support systems can make significant contributions to radiological diagnosis processes with the right model selection and appropriate data preprocessing techniques. Future studies using larger, balanced, and multi-center datasets will increase the generalizability and clinical reliability of the developed models.

## Data Availability

The raw data supporting the conclusions of this article will be made available by the authors, without undue reservation.
